# Predictors of HIV infection and prevalence for syphilis infection among injection drug users in China: Community-based surveys along major drug trafficking routes

**DOI:** 10.1186/1477-7517-5-29

**Published:** 2008-08-25

**Authors:** Yujiang Jia, Fan Lu, Gang Zeng, Xinhua Sun, Yan Xiao, Lin Lu, Wei Liu, Mingjian Ni, Shuquan Qu, Chunmei Li, Jianbo Liu, Pingsheng Wu, Sten H Vermund

**Affiliations:** 1 Institute for Global Health, Vanderbilt University School of Medicine, Nashville, TN 37232, USA; 2 Department of Pediatrics, Vanderbilt University School of Medicine, Nashville, TN 37232, USA; 3 Biostatistics, Vanderbilt University School of Medicine, Nashville, TN 37232, USA; 4 National Center for AIDS Control and Prevention, China center for Disease Control and Prevention, Beijing 100050, PR China; 5 Division of AIDS, Disease Control Bureau, Ministry of Health, Beijing 051000, PR China; 6 Yunnan Center for Disease Control and Prevention, Kunming, Yunnan Province 650032, PR China; 7 Guangxi Zhuang Autonomous Region Center for Disease Control and Prevention, Nanning, Guangxi Zhuang Autonomous Region 530021, PR China; 8 Xinjiang Uygar Autonomous Region Center for Disease Control and Prevention, Urumqi, Xinjiang Uygar Autonomous Region 830002, PR China; 9 Department of Epidemiology and Biostatistics, School of Public Health, Hebei Medical University, Shijiazhuang, Hebei Province 051000, PR China

## Abstract

**Objective:**

To assess the predictors and prevalence of HIV infection among injection drug users in highly endemic regions along major drug trafficking routes in three Chinese provinces.

**Methods:**

We enrolled participants using community outreach and peer referrals. uestionnaire-based interviews provided demographic, drug use, and sexual behavior information. HIV was tested via ELISA and syphilis by RPR.

**Results:**

Of the 689 participants, 51.8% were HIV-infected, with persons living in Guangxi having significantly lower prevalence (16.4%) than those from Xinjiang and Yunnan (66.8% and 67.1%, respectively). Syphilis seropositivity was noted in 5.4%. Longer duration of IDU, greater awareness of HIV transmission routes, and living in Xinjiang or Yunnan were associated with HIV seropositivity on multivariable analysis. Independent risk factors differed between sites. In Guangxi, being male and having a longer duration of IDU were independent risk factors for HIV infection; in Xinjiang, older age and sharing needles and/or syringes were independent factors; in Yunnan, more frequent drug injection, greater awareness of HIV transmission routes, and higher income were independent predictors of HIV seropositivity.

**Conclusion:**

Prevalence rates of HIV among IDUs in China are more than two out of three in some venues. Risk factors include longer duration of IDU and needle sharing. Also associated with HIV were factors that may indicate some success in education in higher risk persons, such as higher knowledge. A systemic community-level intervention with respect to evidenced-based, population-level interventions to stem the spread of HIV from IDU in China should include needle exchange, opiate agonist-based drug treatment, condom distribution along with promotion, and advocacy for community-based VCT with bridges to HIV preventive services and care.

## Background

Injection drug use (IDU) represents the largest single cause of HIV transmission in China, accounting for nearly half of the infections at the end of 2005 [1]. Ministry of Public Security data suggest that the number of registered drug users has risen steadily at a rate of about 122% per year, from 70,000 in 1990 to 1.16 million in 2005 [2-4]. The total number, including unregistered drug users, is thought to be much higher, perhaps 3.5 million [5]. China has the second largest estimate (midpoint: 1.9 million) of IDUs worldwide, following only Russia [6]. The first large outbreak of HIV in China was identified in 1989 among injection drug users (IDUs) in Dehong Prefecture of Yunnan Province on the Myanmar (Burma) border in southwest China [7]. The specific HIV subtypes first seen in Dehong spread along drug trafficking routes to IDUs in nearby cities in Yunnan [8,9]. Since then, serious epidemics among IDUs have been identified in Xinjiang (1996), Guangxi and Sichuan (1997), Guangdong (1998), Gansu (1999), and Jiangxi (2000) [10]. The HIV epidemic routes coincided with the major drug trafficking roads from the "Golden Triangle" into China. Molecular epidemiology suggests that the major spread of the initial drug-related epidemic in China started in Yunnan and took two major routes: northbound to Sichuan, Guizhou, Gansu, Ningxia and Xinjiang, and eastbound to Guangxi, Guangdong and Guizhou [8,9,11-18]. Before 1993, the HIV-infected cases in China were reported mainly from Yunnan [7].

Xinjiang and Sichuan first reported HIV infections among drug users in 1995; the HIV epidemic was first detected among drug users in Guangxi in 1996. In subsequent years, HIV spread rapidly among IDUs in Yunnan, Xinjiang, and Guangxi and by the end of 2002, all 31 provinces, municipalities and autonomous regions in mainland China, as well as Hong Kong, Macao, and Taiwan, had reported cases of HIV/AIDS among drug users from 1989 to 2004. Yunnan reported the highest number of annual HIV/AIDS cases in mainland China [7].

Yunnan's proximity to one of the world's largest illicit drug (especially heroin) production and distribution centers, the "Golden Triangle", contributes to drug trafficking and the availability of heroin [12,19,20]. Only a small portion of heroin/opium is trafficked into Xinjiang from the "Golden Crescent" [3]. Currently, Yunnan, Xinjiang and Guangxi have remained the top three of the hardest-hit regions fueled by IDU in China [7,12,14,18,21-23]. However, no systematic community-based interventions have been undertaken in these regions. Only a small fraction of IDUs receive counseling and testing services and even fewer have participated in methadone maintenance treatment and needle exchange programs that were started in 2004. Several studies have described the different HIV transmission risk factors among IDUs based in detoxification and detention centers in China [24,25]. However, there are few studies that used community-based recruitment of IDUs from multiple provinces [15]. A behavioral survey among drug users in Yunnan, Xinjiang, Hubei, and Beijing found that most of the drug users reported behaviors associated with high rates of HIV/AIDS acquisition, such as unsafe sexual practices and using drugs intravenously (70.6%) [23]. Of those who used drugs intravenously, 89.2% reported sharing needles. The general knowledge about HIV/AIDS among this group was relatively poor. In order to understand the threat of HIV epidemic expansion and guide appropriate HIV prevention among IDUs in three highly endemic regions along drug trafficking routes in China, we conducted this community-based survey to assess the prevalence of HIV and syphilis and predictors for HIV infections.

## Methods

### Study sites

**Figure 1:**
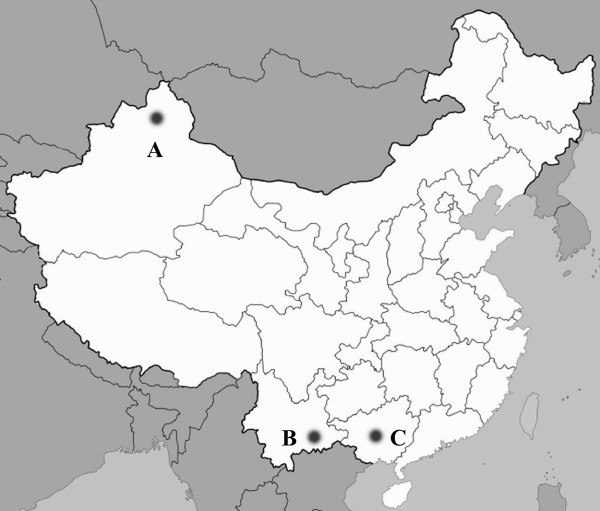
**Location of study sites.** This study was conducted in three sites along major drug (heroin) trafficking routes in Yili Prefecture, Xinjiang Uygar Autonomous Region (A); Honghe Autonomous Prefecture, Yunnan Province (B), and Nanning City, Guangxi Zhuang Autonomous Region (C).

This study was conducted in three sites along major drug (heroin) trafficking routes in Nanning City, Guangxi Zhuang Autonomous Region; Yili Prefecture, Xinjiang Uygar Autonomous Region; and Honghe Prefecture, Yunnan Province (Fig. 1). We chose these three drug trafficking routes/provinces because HIV epidemics in these areas shared certain characteristics. All three regions were hardest hit by HIV, IDU has been the predominant route of transmission for HIV, and non-Han minority ethnic groups account for a large portion of the IDUs. Most of these IDUs live in relatively poor socioeconomic conditions. Guangxi, located along the major drug trafficking trade route bordering Yunnan on the west and Vietnam on the southwest, hosts 49 million people. Nanning is Guangxi's capital city and has a population of almost 2 million, 36% of whom belong to Zhuang ethnic and other non-Han minority ethnic groups. Xinjiang covers a very large area, with 19 million people in far northwestern China, and has the longest boundary in China. From the northeast to the southwest, Xinjiang borders eight countries: Mongolia, Russia, Kazakhstan, Kirghizstan, Tajikistan, Afghanistan, Pakistan, and India. Yili Prefecture, located in the northwest of Xinjiang, hosts 2 million people: 45.2% Han, 25.4% Kazak, 15.9% Uygar, and 13.5% belong to other minorities. Yunnan is located in southwestern China and borders Myanmar, Laos, and Vietnam. Ethnic minorities account for 33.4% of Yunnan's population of 43 million. Honghe Prefecture is located in the south of Yunnan Province. The population of Honghe is about 4.1 million and 40.0% belong to Hani and Yi ethnic groups, while 14.7% belong to other non-Han minorities.

### Study design and study population

Community-based surveys were completed from November 2004 to January 2005. The size of the IDU population was estimated in each community and geographic mapping was conducted for each site in the study's targeted communities. The participants were primarily enrolled by the trained staff using community outreach and peer referral "snowball" techniques. The peer referrals were limited to a maximum of five participants in order to enroll a relatively representative sample in the IDU community. Eligibility criteria required that participants be ≥18 years old and have injected drugs at least one time in the last three months. Blood was collected for HIV and syphilis testing. All eligible participants were provided with risk reduction and coping counseling, both pre- and post-test. Written informed consent was received for all participants. Survey information was collected anonymously and remained confidential. The surveys also served as part of ongoing comprehensive IDU-focused surveillance activity, combining behavioral and biological information [26]. The study was approved by the Institutional Review Board (IRB) of the National Center for AIDS/STD Control and Prevention of the China Centers for Disease Control and Prevention, as well as the IRB of Vanderbilt University.

### Measures

Participants were recruited and completed all study procedures in either Chinese and/or the local language. All interviews were conducted by trained staff in both Chinese and the local languages to provide information including (Table 1 and 2): (1) demographic characteristics, e.g., sex, age, marital status, residency, ethnicity, years of education, monthly income, and study site; (2) drug use behaviors, e.g., duration of drug use, frequency of injecting drugs in the last week, ever shared needle and/or syringe during injection, the number of people shared needle and/or syringe with in the last injection, frequency of shared injection needle and/or syringe in the last six months, always carried a needle and syringe when out, and how many times a needle and syringe was used before trashing it; and (3) sexual behaviors, e.g., living with regular sex partners in the last year, ever had sex with regular sex partner in the last year, condom use with regulars sex partners in the last sex act, frequency of condom use with regular sex partners in the last year, regular sex partners ever used drugs, regular sex partners knew you used drugs, shared needle and/or syringe with regular sex partners, ever had sex with non-regular sex partners in the last year, the number of non-regular sex partners in the last year, condom use with non-regular sex partners in the last sex act, frequency of condom use with non-regular sex partners in the last year, ever paid money or provided drugs for sex in the last year, the number of sex partners paid or provided drugs for sex in the last year, condom use during paid or provided drugs for sex in the last sex act, frequency of condom use during paid or provided drugs for sex in the last year, ever provided sex for money or drugs for sex in the last year, the number of sex partners who had sex for money or drugs in the last year, condom use during sex for money or drugs in the last sex act, and frequency of condom use during sex for money or drugs in the last year. Knowledge about risk of HIV transmission routes was assessed by correctly answering five questions that were related to modes of HIV transmission (blood, sex, and mother to infant). The participants were further asked whether they had ever received voluntary HIV counseling and testing (VCT). All of the above questions in the questionnaire were selected by a panel of consultants of the national behavioral and biological sentinel surveillance in China [26,27].

**Table 1:** Demographic factors associated with HIV infection among injection drug users in three highly endemic regions of China

Factors	N*	% (HIV+)^†^	OR (95% CI)	*P*
Sex				
Female	122	34.4(42)	1.0	<0.001
Male	560	55.4(310)	2.4(1.6–3.6)	
Age				
<30 years	234	462(108)	1.0	0.3
≥ 30 years	353	50.4(178)	1.2(0.9–1.7)	
Marital status				
Married	195	51.8(101)	1.0	0.06
Single	395	49.1(194)	0.9(0.7–1.3)	0.5
Separated	80	63.8(51)	1.6(1.0–2.8)	0.05
Residency				
Local	658	52.7(347)	1.0	0.005
Other province	17	11.8(2)	0.1(0.03–0.5)	
Ethnicity				
Han	390	48.7(190)	1.0	0.03
Other	285	57.2(163)	1.4(1.0–1.9)	
Years of education				
>6 years	487	52.2(254)	1.0	1.0
≤ 6 years	186	52.2(97)	1.0(0.7–1.4)	
Monthly income				
≤ 300 Yuan	220	50.9(112)	1.0	0.08
>300 Yuan	260	58.8(153)	1.4(1.0–2.0)	
District				
Nanning, Guangxi	207	16.4(34)	1.0	<0.001
Yili, Xinjiang	205	66.8(137)	10.3(6.4–16.4)	<0.001
Honghe, Yunnan	277	67.1(186)	10.4(6.7–16.2)	<0.001

*: Total N may not equal 689, because of missing data; N: the number of participants being tested; ^†^: %: the prevalence of HIV infection; HIV+: the number of HIV positive.

**Table 2:** Factors associated with HIV infection among injection drug users in three highly endemic regions of China


Factors	N*	% (HIV+)^†^	OR (95% CI)	P

**Knowledge of three major transmission routes for HIV**				
Yes	548	53.8 (295)	1.0	0.006
No	129	40.3 (52)	0.6 (0.4–0.9)	
**Received voluntary counseling and testing**				
No	628	51.3 (322)	1.0	0.03
Yes	34	70.6 (24)	2.5 (1.2–5.0)	
**Drug use behaviors**				
Duration of drug use (injection plus non-injection)				
<5 years	114	37.7 (43)	1.0	0.002
≥ 5 years	432	53.9 (233)	1.9 (1.3–3.0)	
Duration of injection drug use				
<5 years	186	34.9 (65)	1.0	0.001
≥ 5 years	381	56.7 (216)	2.4 (1.7–3.5)	
Frequency of drug injection in the last week				
<2 times	470	53.8 (253)	1.0	0.6
≥ 2 times	176	51.7 (91)	0.9 (0.6–1.3)	
Ever shared needle and/or syringe during injection				
No	328	49.4 (162)	1.0	0.2
Yes	341	54.8 (187)	1.2 (0.9–1.7)	
Ever shared needle and/or syringe in the last injection				
No	586	51.2 (300)	1.0	0.1
Yes	78	60.3 (47)	1.4 (0.9–2.3)	
No. of people shared needle and/or syringe in the last injection				
= 1	19	47.4(19)	1.0	0.3
>1	56	62.5(35)	1.8(0.6–5.3)	
Frequency of shared injection needle and/or syringe in the last 6 months				
Never	516	52.3(516)	1.0	0.4
Sometimes	137	51.8(137)	1.0(0.7–1.4)	0.9
Always	6	83.3(5)	4.6(0.5–39.3)	0.2
Always carried a needle and syringe with you when you were out				
Yes	171	53.8(92)	1.0	0.4
No	409	57.5(235)	1.2(0.8–1.7)	
How many times a needle and syringe was used before trashing it				
1 times	252	59.1(149)	1.0	
2 times	194	56.7(110)	0.9(0.6–1.3)	0.7
>2 times	105	48.6(51)	0.7(0.4–1.0)	0.08
**Sexual behavior**				
Living with regular sex partners in the last year				
Yes	282	51.8 (146)	1.0	0.8
No	380	52.9 (201)	1.0(0.8–1.4)	
Ever had sex with regular sex partners in the last year				
Yes	257	51.8(133)	1.0	0.8
No	23	52.2(12)	1.0 (0.4–2.4)	
Condom use with regular partner in the last sex act				
Yes	76	52.6(40)	1.0	0.8
No	186	51.1(95)	0.9(0.6–1.6)	
Frequency of condom use with regular sex partner in the last year				
Always	81	45.7 (37)	1.0	0.4
Sometimes	146	54.8 (80)	1.4(0.8–2.5)	0.2
Never	32	53.1 (17)	1.3(0.6–3.1)	0.5
Regular sex partners ever used drugs				
No	199	49.7 (99)	1.0	0.6
Yes	95	52.6 (50)	1.1 (0.7–1.8)	
Regular sex partner knew you used drugs				
No	81	48.1(39)	1.0	0.6
Yes	214	51.4(110)	1.1(0.7–1.9)	
Ever shared needle and/or syringe with a regular sex partner				
No	47	53.2(25)	1.0	0.9
Yes	46	54.3(25)	1.0(0.5–2.4)	
Ever had sex with non-regular partners in the last year				
No	528	53.4 (282)	1.0	0.3
Yes	132	48.5 (64)	0.8 (0.6–1.2)	
No. of non-regular sex partners in the last year				
1	45	51.1(23)	1.0	0.5
>1	56	44.6(25)	0.8(0.4–1.7)	
Condom use with non-regular sex partner in the last sex act				
Yes	42	54.8(23)	1.0	0.3
No	92	45.7(42)	0.7(0.3–1.4)	
Frequency of condom use with non-regular sex partners in the last year				
Always	73	39.7 (29)	1.0	0.1
Sometimes	36	58.3 (21)	2.1(0.9–4.8)	0.1
Never	24	58.3 (14)	2.1(0.8–5.4)	0.2
Ever paid or provided drugs for sex in the last year				
No	507	56.2(507)		0.2
Yes	41	43.9(41)	0.6(0.3–1.2)	
No. of sex partners ever paid or provided drugs for sex in the last year				
1	10	50.0(5)	1.0	0.8
>1	24	45.8(11)	0.8(0.2–3.7)	
Condom use during paid or provided drugs for sex in the last sex act				
Yes	13	53.8(7)	1	0.3
No	28	39.3(11)	0.4(0.1–1.8)	
Frequency of condom use during paid or provided drugs for sex in the last year				
Always	5	60.0(3)	1	0.2
Sometimes	12	66.7(8)	0.4(0.2–11.5)	1
Never	23	30.4(7)	0.1(0.04–2.1)	0.3
Ever provided sex for money or drugs in the last year				
No	83	32.4(27)		0.7
Yes	34	38.7(13)	1.2(0.6–2.9)	
No. of sex partners for money or drugs in the last year				
1	0	0	-	
>1	19	36.8(8)	-	
Condom use during sex for money or drugs in the sex act				
Yes	13	30.8(4)		0.7
No	16	43.8(7)	1.8(0.4–8.1)	
Frequency of condom use during sex for money or drugs in the last year				
Always	0	0	-	
Sometimes	5	60.0(3)	-	
Never	7	14.3(1)	-	
**Syphilis sero-status**				
Negative	614	50.0(307)	1.0	0.9
Positive	33	48.5(16)	0.9(0.5–1.9)	

*: Total N may not equal 689, because of missing data; N: the number of participants being tested for HIV; ^†^: %: the prevalence of HIV infection; HIV+: the number of HIV positive.

All collected serospecimens were stored at the Prefecture-level CDC laboratories and transported to Provincial-level CDC for HIV testing. Two Enzyme-Linked ImmunoSorbent Assays (ELISA, Vironostika HIV Uni-form II plus O™, BioMérieux, Marcy L'Etoile, France; Beijing Wantai Biologic Medicine Co., China) were performed. Both samples testing positive were considered HIV-positive; both samples testing negative were considered HIV-negative. A repeat second ELISA was used as a tiebreaker for discordant results. Western blot confirmation of cases was possible in one province consistently, one province intermittently, but was not used in the third province. Syphilis serostatus was determined by screening for the antibody to *Treponema pallidum *antigen (p15, p17, and p47) and by a positive rapid-plasma reagin (RPR) test (Macro-Vue RPR™ Card Test, Becton-Dickinson, USA).

### Statistical analysis

Data were entered with EpiData. After corrections, data were then converted and analyzed using the Statistical Package for the Social Sciences (SPSS 15 for Windows™; SPSS Inc., Chicago, Il, USA). The data were analyzed using unadjusted odds ratios with 95% confidence intervals for the odds ratio point estimates. Tests for significance of categorical data used a Chi-square test or Fisher's exact test. A multivariable logistic regression model was constructed with all variables in the univariate model whose p value was less than 0.2. Thus, we report independent risk factors for HIV infection, controlling for confounding and interaction from other putative risk factors.

## Results

### Socio-demographic characteristics

We included 689 eligible participants (95.4%) for the analyses; 33 persons were excluded because of refusing to participate or not meeting eligibility criteria. Of the participants, 82.0% were males; 53.8% were of the majority Han ethnicity; 72.4% had <6 years of education; and 59.0% were single, 29.1% married, and 11.9% separated (Table 1). Their average age was 30.8 years old (S.D. ± 6.0) and 40.0% were under 30 years old; 97.5% were local residents; and 54.2% had ≤ 300 Yuan monthly incomes (Table 1).

### HIV knowledge and VCT

Of the participants, 80.9% were aware of all three transmission routes (blood, sex, and mother-to-child); only 5.1% of the participants had ever received VCT (Table 2).

### Drug use and sexual behaviors

Of the participants, 79.1% had used illicit drugs >5 years; 79.1% injected drugs for ≥ 5 years; and 51.0% reported a history of sharing needles and/or syringes. To judge current users, we determined that 27.2% had injected drugs more than twice in the prior week. Of the 11.7% participants who reported using a shared needle and/or syringe in the last injection, three-quarters of them shared with more than one person. Of the participants, 70.5% reported never carrying a needle and syringe when they were out. 54.3% of the participants reported used a needle and syringe more than once before trashing it. One-fifth of participants reportedly had sex with non-regular partners in the last year. One-third of subjects reported always using condoms when having sex with their regular partner in the last year, while 40.0% reported always using condoms when having sex with non-regular sex partners in the last year. Over the last year, 7.5% had paid money or provided drugs for sex and only 12.5% of them reported using condoms consistently. 29.1% provided sex for money or drugs and none of them reported using condoms consistently (Table 2).

### Prevalence of syphilis seropositivity and predictors for HIV seropositivity

Of the 689 participants, 5.4% were RPR reactive for syphilis. 51.8% were HIV-seropositive, with persons living in Guangxi having significantly lower prevalence (16.4%) than those from Xinjiang and Yunnan (66.8% and 67.1%, respectively). In univariate analyses, risk factors associated with HIV sero-positive status included male sex, "separated" marital status, local residency, minority (i.e., non-Han) ethnicity, study site (Yili, Xinjiang and Honghe, Yunnan), awareness of HIV transmission routes, having received VCT, longer duration of drug use, and longer duration of IDU (Table 1 and 2). Sexually-related factors, age, years of education, and syphilis seropositivity were not associated significantly with HIV seropositive status.

Multivariable logistic regression analyses suggested that a longer duration of IDU (Adjusted OR = 3.5; 95%CI: 1.4–8.5), greater awareness of HIV transmission routes (AOR = 2.0; 95%CI: 1.0–3.3), and living in Yili, Xijiang (AOR = 7.7; 95%CI: 4.4–13.4) and Honghe, Yunnan (AOR = 15.1; 95%CI: 7.2–31.7) versus in Nanning, Guangxi, were independent risk factors for HIV sero-positivity for the three sites (Table 3). In Nanning, Guangxi, being male (AOR = 3.3; 95%CI: 1.1–10.2) and having a longer duration of IDU (AOR = 4.5; 95%CI: 1.3–15.6) were independent risk factors for HIV sero-positive. In Yili, Xinjiang, older age (AOR = 3.7; 95%CI: 1.2–11.8) and ever sharing of needles and/or syringes (AOR = 5.7; 95% CI: 1.1–29.1) were independent risk factors for HIV sero-positive. In Honghe, Yunnan, higher frequency of drug injection (AOR = 3.7; 95%CI: 1.5–8.7), greater awareness of HIV transmission routes (AOR = 2.5; 95%CI: 1.0–6.0), and higher income (AOR = 1.8; 95% CI: 1.0–3.4) were independent risk factors for HIV sero-positive status.

**Table 3:** Factors associated with HIV infection among injection drug users in three highly endemic regions of China, as predicted by a multivariable logistic regression model

Factors	AOR (95% CI)	*P*
Three sites		
Duration of injection drug use: ≥ 5 years versus <5 years	3.5 (1.4–8.5)	<0.01
Awareness of HIV transmission: awareness vs. unawareness	2.0 (1.0–3.3)	<0.05
Yili Prefecture, Xinjiang: vs. Nanning, Guangxi	7.7 (4.4–13.4)	<0.001
Honghe Prefecture, Yunnan: vs. Nanning, Guangxi	15.1 (7.2–31.7)	<0.001
Site 1, Nanning, Guangxi		
Duration of injection drug use: ≥ 5 years vs. <5 years	4.5 (1.3–15.6)	0.02
Sex: Male vs. female	3.3 (1.1–10.2)	0.04
Site 2, Yili, Xinjiang		
Shared injection needle and/or syringe: Yes vs. No	5.7 (1.1–29.1)	0.04
Age: ≥ 30 years old vs. <30 years old	3.7 (1.2–11.8)	0.03
Site 3, Honghe, Yunnan		
Frequent drug injection: 0–1 time/week vs. ≥ 2 times/week	3.7 (1.5–8.7)	<0.001
Awareness of HIV transmission: awareness vs. unawareness	2.5 (1.0–6.0)	0.05
Monthly income: ≥ 300 Yuan vs. <300 Yuan	1.8 (1.0–3.4)	<0.05

AOR = Adjusted Odds Ratio

## Discussion

We assessed the prevalence and predictors of HIV sero-positive among 689 IDUs with serious illicit drug problems in China using community-based cross sectional surveys with consistent sampling procedures in all three provinces (or autonomous regions). HIV prevalence was very high (51.8%), but was lower in persons living in Guangxi (16.4%) compared to Xinjiang and Yunnan (67.8%). The HIV prevalence rates were remarkably similar to those from the same sites among IDUs from detoxification or detention centers [22], and were significantly higher than estimates from community-based surveys in other regions in China [27].

Lower rates are reported in other provinces. For example, in January 2005, HIV prevalence rates of 0% to 5.9% were reported in six community-based surveys of 1,260 IDUs in Guangxi and Yunnan's adjacent provinces of Sichuan (3.7%), Guangdong (5.9%), and Guizhou (0%), with even lower prevalence noted in sites in Fujian (0.4%), Henan (0%), and Hubei (0%), provinces located farther from Guangxi and Yunnan [27]. Higher HIV prevalence rates among IDUs in 2004–2006 surveys are seen in those regions of Guangxi, Xinjiang, and Yunnan where rapid spread of the virus among drug users occurred earliest; HIV was first reported in Yunnan in 1989 [7,22]. Overall prevalence was noted to be 71.9% among IDUs from detoxification centers in Honghe and Wenshan Prefectures of Yunnan Province in 2000, having declined subsequently. One may speculate that rates have dropped due to deaths and/or prevention successes [28]. Five out of 15 prefectures in Yunnan have reported high HIV prevalence rates among IDUs, ranging from 48.9% to 75.0% [7,22,29]. Biological sentinel surveillance data show that HIV prevalence rates have reached 75.0% in certain sites of Xinjiang and 51.0% in certain sites of Guangxi in 2005 [22]. The majority of the participants in sentinel surveillance were recruited from detoxification or detention centers and they are likely to be higher risk injectors than IDUs in community settings. These differences could also reflect the availability of proactive testing in the detoxification or detention centers rather than a proven difference between the sub-group and a wider population of IDUs.

High HIV prevalence among IDUs, prevalent needle sharing and high frequency of injecting practices suggest an urgent need to improve drug addiction treatment and risk reduction measures in China. We found that 51.0% of the participants had shared needles and/or syringes and 27.2% had injected drugs more than twice in the last week. An HIV epidemic becomes self-perpetuating (endemic) and even a modest level of risk behavior can lead to a substantial rate of infection in the face of efficient needle/blood transmission [30,31]. Because they live along major drug trafficking routes, many of the HIV-infected IDUs in our survey will continue to serve as a major source for continued transmission and further spread unless drug abuse treatment, antiretroviral therapy, and risk reduction are implemented, as indicated[32].

While longer duration of IDU, shared injection needle and/or syringe, and higher frequency of injection were the independent risk factors for HIV infection [14,15,33,34], greater awareness of HIV was associated (unexpectedly) with higher HIV prevalence. This may suggest some successes in educating IDUs. Higher income was also a risk factor. We speculate that drug users with higher incomes may use drugs more often; they may also have a greater awareness of HIV issues. There was some diversity in associated risk factors among the IDU subgroups in the three regions where HIV prevalence was especially high. Although a high portion of participants know HIV transmission routes in all three sites, the needle sharing rates and unprotected sexual behaviors were still high among IDUs. Most astonishingly, a very small portion (overall 5.1%) of participants reported ever receiving VCT, a gateway for the prevention programs. This indicated that a large proportion of IDUs who have been infected with HIV don't know their status and could continue to spread the virus [26,35]. China has scaled up HIV control efforts since 2004 [35]; however, low HIV testing rates (≈20% nationwide) remain an impediment to prevention and care. Lack of affordable accessibility to sterile needles and syringes was the major reason for high risk sharing of "works" in this study. Other data suggested social norms that foster stigma, discrimination associated with drug use and HIV/AIDS, fear of arrest due to illegal practice, knowing a positive result, a lack of coping skills, and knowledge of HIV risks are the other reasons for the low rate of HIV testing among IDUs [4,26]. This suggested that risk reduction education alone cannot help drug users and their sex partners make lasting behavioral changes. The community-based needle exchange programs and elimination of any barriers to accessing clean needles and syringes could reduce the prevalence of needle sharing among IDUs[36,37]. In addition to providing accurate and up-to-date information on risky behaviors, effective community-based prevention programs not only make clean needles and condoms available and accessible, but also focus on enhancing individuals' motivation to change their behavioral patterns, teaching concrete strategies, and behavioral skills to reduce risk, providing tools for risk reduction, and reinforcing positive behavior change.

We found that there were significant differences between sex, age, marital status, residency, ethnicity, education level, and monthly income among the participants in the three study sites. A larger portion of participants who were single and belong to the Han ethnic group, with >6 years of education and higher income, were recruited in Honghe, Yunnan than in the other two sites. Yili, Xijiang's participants were more likely to be younger, belong to non-Han ethnic groups (86.9% Wei ethnic group in Yili, Xijiang; 11.2% Hani and Yi ethnic groups in Honghe, Yunnan and 32.2% Zhuang ethnic group in Nanning, Guangxi), and receive lower levels of education. Nanning, Guangxi's participants were more likely to have less monthly income (74.2% with ≤ 300 Yuan RMB monthly income). We found that higher income in Honghe, being male in Nanning, and old age in Yili were independently associated with HIV infection. There could be other factors beyond this study, besides gender, age and the sharing of needles, such as the actual availability of syringe distribution and exchange programs, condom distribution and promotion, and other social determinants of health that account for the differences for the HIV prevalence rates in the three study sites. China's central government has scaled up HIV/AIDS control efforts since 2004 [35], including setting up national policy framework for responding to HIV/AIDS, increasing funding inputs, and expanding collaborations with international organizations. However, responses to drug use and the HIV/AIDS epidemic vary significantly at provincial and lower administrative levels. A literature review indicated that Yunnan and Guangxi provinces have done far more than other provinces in supporting, implementing, and advocating for harm reduction interventions for IDUs [4]. Some local governments are not fully motivated to confront drug abuse and HIV/AIDS problems [4].

Among IDUs in other studies from China, risky sexual behaviors have been reported as a risk factor for HIV infection [14,15,34], although we did not find this association in our three populations. Most of our participants that lived in remote rural areas of Honghe, Yunnan and Yili, Xinjiang were less likely to receive health education and services. Furthermore, due to relatively poor economic status and lower levels of education, they may be more likely to be involved in drug smuggling and abuse, and unprotected sexual behavior. Risk reduction programs should give high priority to these poorer, more isolated IDUs who are also more likely to be of minority ethnic origin. Because of the high prevalence of HIV and often risky sexual behavior among IDUs, there is a great potential for IDUs serving as a bridge population to transmit HIV to the general population. The overlapping of risk behaviors among at-risk persons facilitates the rapid HIV spread from IDUs to other risk groups, e.g., from female sex workers and their clients to their clients' regular partners. We found that low condom use rates and the high proportion of female drug users who had reported engaging in commercial sex underscore the importance of behavioral surveillance in IDUs to provide early warnings and more effective interventions. This highlighted the need for condom distribution and promotion. As noted in this study, most of the target IDUs interviewed already knew the causes of HIV; the problem is not knowledge translation, it is more basic social determinants of health. They don't have access to free condoms. Free condoms should be provided widely to sex trade workers and IDUs.

The prevalence of syphilis by RPR in our high risk IDUs was 5.4% (33/647), similar to estimates in 10 sentinel surveillance sites using RPR screening in 1,414 IDUs in the same three provinces (average: 6.6%, range from 1.2 to 14.1%) [22]. Syphilis seropositivity did not predict HIV, suggesting that most infections were due to injection-related behaviors. Other studies have reported an association between HIV infection and other STDs among IDUs [38-41]. Syphilis should be considered one indicator of high sexual risk behavior among IDUs [42]. Previous studies of syphilis among IDUs have suggested that while a high prevalence of syphilis and low HIV prevalence may be found in clinical or community settings, the reverse pattern of high HIV prevalence and low prevalence of syphilis may be more common in detoxification centers where IDUs, who are heavier drug users, are overrepresented [22,43]. The patterns of STD co-morbidity among IDUs vary significantly by venue and high risk group [22,44].

Strengths of this study include its substantial sample size, the geographic diversity of our venues, and community-based outreach and peer referral using "snowball" and mapping strategies. There are also limitations. First, IDUs recruited into the study may have been higher risk such that their HIV prevalence may not exactly reflect the true background rate among IDUs in the study community. Second, recall bias and social desirability bias are possible, since the drug use and sexual behavioral information was collected based on self-reporting. Most information about drug use and sexual behaviors in the last year were used in the data collection, instead of collecting the behaviors in more recent period, in the last three or six months. Third, our cross-sectional study cannot ascertain a causal association between predictors and HIV infections. Fourth, we do not include a complete list of factors in this study. Other factors beyond this study may also account for the differences.

China has initiated harm reduction projects, including needle exchange programs, methadone treatment, condom promotion, and VCT programs among drug users [4,25,36,37,45,46]. China Center for Disease Control and Prevention provincial authorities have been organizing the needle exchange and methadone treatments since early 2004 [20,46,47]. China plans to scale up harm reduction projects, including needle exchange programs and methadone treatments, since only a small portion of IDUs have been covered by these programs so far. Our data suggest the urgent need for expanded community-level needle exchange programs, opiate agonist-based drug treatment, and advocacy for community-based VCT with bridges to HIV preventive services and care. Condom distribution along with condom promotion should also be highlighted. In vulnerable target populations where condom use is directly related to availability, condom distribution and promotion is crucial to helping curb the spread of HIV and other STDs. These prevention and treatment efforts are likely to require an infrastructure that not only provides operational and financial support, but also creates an environment in which IDUs feel comfortable and safe in seeking help without any barriers. Implementation research programs can critically assess these programs and provide insight as to where they might be improved.

## Competing interests

The authors declare that they have no competing interests.

## Authors' contributions

YJ participated in the development of the manuscript, coordinated the analysis, and drafted the manuscript. FL, ZG, and XS were responsible for securing funding, supervising data collection, and preparation of the manuscript. YX provided data analysis, and drafted and reviewed the manuscript. CL and PW served as the statisticians for the manuscript. LW, LL, MN, and SQ oversaw all recruitment efforts in the field, supervised HIV and syphilis tests, and were an active part of the preparation of the manuscript. SHV provided input with guidance on the data analysis and interpretation, and co-wrote the manuscript. All authors read and approved the final manuscript.

## References

[B1] Lu F, Wang N, Wu Z, Sun X, Rehnstrom J, Poundstone K, Yu W, Pisani E (2006). Estimating the number of people at risk for and living with HIV in China in 2005: methods and results. Sex Transm Infect.

[B2] Kulsudjarit K (2004). Drug problem in southeast and southwest Asia. Ann N Y Acad Sci.

[B3] Publication UN, UNODC (2007). 2007 World drug report.

[B4] Qian HZ, Schumacher JE, Chen HT, Ruan YH (2006). Injection drug use and HIV/AIDS in China: review of current situation, prevention and policy implications. Harm Reduct J.

[B5] Lu L, Fang Y, Wang X (2008). Drug Abuse in China: Past, Present and Future. Cell Mol Neurobiol.

[B6] Aceijas C, Friedman SR, Cooper HL, Wiessing L, Stimson GV, Hickman M (2006). Estimates of injecting drug users at the national and local level in developing and transitional countries, and gender and age distribution. Sex Transm Infect.

[B7] Xiao Y, Kristensen S, Sun J, Lu L, Vermund SH (2007). Expansion of HIV/AIDS in China: lessons from Yunnan Province. Soc Sci Med.

[B8] Kou J, Cheng H, Zhang J (1997). The overgrowing epidemic of HIV infection in Yunnan Province. Zhouguo Aizibing Xingbing Za Zhi.

[B9] Jia M, Zhang J, Chen H (1999). The epidemic of HIV infection and prevention researches in Yunnan 1989-1998. Zhonghua Liu Xing Bing Xue Za Zhi.

[B10] Yang H, Li X, Stanton B, Liu H, Liu H, Wang N, Fang X, Lin D, Chen X (2005). Heterosexual transmission of HIV in China: a systematic review of behavioral studies in the past two decades. Sex Transm Dis.

[B11] Yu XF, Chen J, Shao Y, Beyrer C, Liu B, Wang Z, Liu W, Yang J, Liang S, Viscidi RP, Gu J, Gurri-Glass G, Lai S (1999). Emerging HIV infections with distinct subtypes of HIV-1 infection among injection drug users from geographically separate locations in Guangxi Province, China. J Acquir Immune Defic Syndr.

[B12] Fu L, Lu L, Jia M, Zhang X, Luo H, Ma Y (2004). Analysis for epidemic trend of acquired immunodeficiency syndrome in Yunnan Province of China. Zhonghua Yu Fang Yi Xue Za Zhi.

[B13] Yang Y, Ma Y, Li Z, Zhang K, Yang W, Ren X (1990). HIV was first discovered among IDUs in China. Zhonghua Liu Xing Bing Xue Za Zhi.

[B14] Liu W, Chen J, Rodolph M, Beauchamp G, Masse B, Wang S, Li R, Ruan Y, Zhou F, Leung MK, Lai S, Shao Y, Jackson JB (2006). HIV prevalence among injection drug users in rural Guangxi China. Addiction.

[B15] Ruan Y, Chen K, Hong K, He Y, Liu S, Zhou F, Qin G, Chen J, Xing H, Shao Y (2004). Community-based survey of HIV transmission modes among intravenous drug users in Sichuan, China. Sex Transm Dis.

[B16] Wei L, Chen J, Rodolph M, Beauchamp G, B MS, Li R, Wang S, Ruan Y, Lai S, Zhang L, Zhou F, Rose SM, Perdue T, Lai S, Shao Y, Jackson JB (2006). HIV Incidence, Retention, and Changes of High-Risk Behaviors Among Rural Injection Drug Users in Guangxi, China. Subst Abus.

[B17] Yin L, Qin GM, Ruan YH, Zhang L, Hao QN, Chen XH, Jiang ZQ, Song BL, Liu SZ, Cao XY, Hao C, Chen KL, Shao YM (2006). [A prospective cohort study on human immunodeficiency virus and syphilis seroconversion among injecting drug users]. Zhonghua Liu Xing Bing Xue Za Zhi.

[B18] Zhang Y, Shan H, Trizzino J, Ruan Y, Beauchamp G, Masse B, Ma J, Gu Y, He Y, Rui B, Wang J, Poundstone K, Jiang Y, Brooks Jackson J, Shao Y (2007). Demographic characteristics and risk behaviors associated with HIV positive injecting drug users in Xinjiang, China. J Infect.

[B19] Wu Z (1998). Recent trends of injecting drug use and related HIV infection in China. Global Research Network Meeting on HIV Prevention in Drug-Using Populations, Inauguration Meeting Report. National Institute on Drug Abuse, 1999.

[B20] Cohen J (2004). HIV/AIDS in China. Changing course to break the HIV-heroin connection. Science.

[B21] Jia Y, Sun J, Lu F, Song D, Tian SM, Yang YC, Lu L, Jia MH, Sun XH, Zhang SG, Kulczycki A, Vermund SH (2008). Estimates of HIV prevalence in a highly endemic area of China: Dehong Prefecture, Yunnan Province. Int J Epidemiol (in revision).

[B22] China CDC (2005). National HIV/AIDS Surveillance Report.

[B23] L. G, Liu ZM, Lian Z, Mu Y, Zhou WH, Wang ZY (2001). Knowledge and risk behaviour on HIV/AIDS among drug users in four areas in China. Chin J Drug Depend.

[B24] Chen HT, Liao Q (2005). A pilot study of the NGO-based relational intervention model for HIV prevention among drug users in China. AIDS Educ Prev.

[B25] Qian HZ, Hao C, Ruan Y, Cassell HM, Chen K, Qin G, Yin L, Schumacher JE, Liang S, Shao Y (2008). Impact of methadone on drug use and risky sex in China. J Subst Abuse Treat.

[B26] Jia Y, Lu F, Sun X, Vermund SH (2007). Sources of data for improved surveillance of HIV/AIDS in China. Southeast Asian J Trop Med Public Health.

[B27] China CDC (2005). National HIV/AIDS biological and behavioral surveillance report.

[B28] Zhang C, Yang R, Xia X, Qin S, Dai J, Zhang Z, Peng Z, Wei T, Liu H, Pu D, Luo J, Takebe Y, Ben K (2002). High prevalence of HIV-1 and hepatitis C virus coinfection among injection drug users in the southeastern region of Yunnan, China. J Acquir Immune Defic Syndr.

[B29] SF. P, Chen HH, Zhang JP (2000). Analysis and prediction on trends of HIV infection epidemic in Yunnan Province. Chin J AIDS/STD Control and Prevention.

[B30] Friedman SR, Jose B, Deren S, Des Jarlais DC, Neaigus A (1995). Risk factors for human immunodeficiency virus seroconversion among out-of-treatment drug injectors in high and low seroprevalence cities. The National AIDS Research Consortium. Am J Epidemiol.

[B31] Des Jarlais C, Perlis T, Friedman SR, Chapman T, Kwok J, Rockwell R, Paone D, Milliken J, Monterroso E (2000). Behavioral risk reduction in a declining HIV epidemic: injection drug users in New York City, 1990-1997. Am J Public Health.

[B32] Pang L, Hao Y, Mi G, Wang C, Luo W, Rou K, Li J, Wu Z (2007). Effectiveness of first eight methadone maintenance treatment clinics in China. Aids.

[B33] Yin L, Qin G, Qian HZ, Zhu Y, Hu W, Zhang L, Chen K, Wang Y, Liu S, Zhou F, Xing H, Ruan Y, Wang N, Shao Y (2007). Continued spread of HIV among injecting drug users in southern Sichuan Province, China. Harm Reduct J.

[B34] Liu H, Grusky O, Li X, Ma E (2006). Drug users: a potentially important bridge population in the transmission of sexually transmitted diseases, including AIDS, in China. Sex Transm Dis.

[B35] China MOH, UNAIDS, WHO (2006). 2005 Update on the HIV/AIDS Epidemic and Response in China (online available at: http://data.unaids.org/publications/External-Documents/rp_2005chinaestimation_25jan06_en.pdf, accessed on August 6, 2008).

[B36] Liu B, Sullivan SG, Wu Z (2007). An evaluation of needle exchange programmes in China. Aids.

[B37] Wu Z, Luo W, Sullivan SG, Rou K, Lin P, Liu W, Ming Z (2007). Evaluation of a needle social marketing strategy to control HIV among injecting drug users in China. Aids.

[B38] (1988). Continuing increase in infectious syphilis--United States. MMWR Morb Mortal Wkly Rep.

[B39] Levine OS, Vlahov D, Koehler J, Cohn S, Spronk AM, Nelson KE (1995). Seroepidemiology of hepatitis B virus in a population of injecting drug users. Association with drug injection patterns. Am J Epidemiol.

[B40] Onorato IM, Klaskala W, Morgan WM, Withum D (1995). Prevalence, incidence, and risks for HIV-1 infection in female sex workers in Miami, Florida. J Acquir Immune Defic Syndr Hum Retrovirol.

[B41] Zeldis JB, Jain S, Kuramoto IK, Richards C, Sazama K, Samuels S, Holland PV, Flynn N (1992). Seroepidemiology of viral infections among intravenous drug users in northern California. West J Med.

[B42] Quinn TC, Glasser D, Cannon RO, Matuszak DL, Dunning RW, Kline RL, Campbell CH, Israel E, Fauci AS, Hook EW (1988). Human immunodeficiency virus infection among patients attending clinics for sexually transmitted diseases. N Engl J Med.

[B43] Muga R, Roca J, Tor J, Pigem C, Rodriguez R, Egea JM, Vlahov D, Munoz A (1997). Syphilis in injecting drug users: clues for high-risk sexual behaviour in female IDUs. Int J STD AIDS.

[B44] Holmberg SD (1996). The estimated prevalence and incidence of HIV in 96 large US metropolitan areas. Am J Public Health.

[B45] Wu Z, Sun X, Sullivan SG, Detels R (2006). Public health. HIV testing in China. Science.

[B46] Sullivan SG, Wu Z (2007). Rapid scale up of harm reduction in China. Int J Drug Policy.

[B47] Wu Z, Sullivan SG, Wang Y, Rotheram-Borus MJ, Detels R (2007). Evolution of China's response to HIV/AIDS. Lancet.

